# Optimal adjustment of the human circadian clock in the real world

**DOI:** 10.1371/journal.pcbi.1008445

**Published:** 2020-12-28

**Authors:** Samuel Christensen, Yitong Huang, Olivia J. Walch, Daniel B. Forger

**Affiliations:** 1 Department of Mathematics, University of California, Los Angeles, California, United States of America; 2 Department of Mathematics, Dartmouth College, New Hampshire, United States of America; 3 Department of Neurology, University of Michigan, Ann Arbor, Michigan, United States of America; 4 Department of Mathematics, Department of Computational Medicine and Bioinformatics, and Michigan Institute for Data Science, University of Michigan, Ann Arbor, Michigan, United States of America; King’s College London, UNITED KINGDOM

## Abstract

Which suggestions for behavioral modifications, based on mathematical models, are most likely to be followed in the real world? We address this question in the context of human circadian rhythms. Jet lag is a consequence of the misalignment of the body’s internal circadian (~24-hour) clock during an adjustment to a new schedule. Light is the clock’s primary synchronizer. Previous research has used mathematical models to compute light schedules that shift the circadian clock to a new time zone as quickly as possible. How users adjust their behavior when provided with these optimal schedules remains an open question. Here, we report data collected by wearables from more than 100 travelers as they cross time zones using a smartphone app, *Entrain*. We find that people rarely follow the optimal schedules generated through mathematical modeling entirely, but travelers who better followed the optimal schedules reported more positive moods after their trips. Using the data collected, we improve the optimal schedule predictions to accommodate real-world constraints. We also develop a scheduling algorithm that allows for the computation of approximately optimal schedules "on-the-fly" in response to disruptions. User burnout may not be critically important as long as the first parts of a schedule are followed. These results represent a crucial improvement in making the theoretical results of past work viable for practical use and show how theoretical predictions based on known human physiology can be efficiently used in real-world settings.

## Author summary

Jet lag, a significant problem for travelers and shift workers, occurs when our body’s internal circadian (~24-hour) clock is misaligned with the time of day in the environment. Such circadian misalignment can lead to decreased performance, impaired sleep, and increased risk for severe health conditions, ranging from cancer to cardiovascular disease. Previous work has proposed mathematically optimal schedules, based on mathematical models of the human circadian pacemaker, to overcome jet lag in minimal time. Here, we use data collected from over 100 travelers by a mobile app to track when users followed or deviated from optimal schedules. Better adherence to the schedules yielded better outcomes. We also propose more practical schedules, which can be adjusted to the real-world challenges in overcoming jet lag. Our work sets the stage for changing human behaviors in other domains by computing personalized recommendations from mathematical models.

## Introduction

Human internal timekeeping is governed by an internal circadian clock located in the suprachiasmatic nuclei (SCN) [1,2]. Light is often thought of as the most critical signal to this clock [1,3]. Many observed circadian phenomena, including the clock’s ability to both phase advance and delay in response to light, can be explained through the effect of light on the SCN, and these physiological phenomena have been codified in mathematical models [4–7]. Using these models and optimal control techniques, we can compute optimal schedules to phase shift the clock under given constraints. These techniques allow us to find, for instance, the maximum phase shift that can be achieved with light up to maximal light intensity or a pattern of light and dark conditions that achieve a target phase shift in minimum time [8–10]. Previous treatments of optimal circadian control have typically focused more on the mathematical tools used to arrive at the predictions than the practicality of the predictions [9,10]. However, usefulness is crucial, as mathematically optimal recommendations can be difficult or nearly impossible to follow in real life.

Past work on accelerating circadian readjustment has included light pulses [11], amplitude suppression [12], intermittent light [13,14], and avoidance of morning light [15]. However, this work focuses primarily on the method proposed in Serkh and Forger [10], a modification of the Switch Time Optimization method. It seeks to adapt its results for real-world use. In Serkh and Forger [10], hundreds of schedules for phase-shifting the clock as quickly as possible were computed for different phase shifts and five maximum light levels (200, 500, 1000, and 10000 lux). These schedules broadly shared several key features: the optimal control was “bang-bang,” so that schedules could be reduced to times at which the recommendation switches from the brightest light available to darkness and vice-versa; the switch times (i.e., the times at which the light is switched on or off) were relatively infrequent, with roughly one block of light and one block of darkness per 24 hours; and each schedule started at the onset of light in the target time zone. However, no constraints were posed in response to the practicality of the schedules. Schedules computed from the "bang-bang" control might recommend travelers avoid light until 16:00 or 17:00 in the new time zone. This is mathematically optimal, but logistically infeasible for many travelers.

Here, we introduce new approaches for adjusting the circadian clock to a new time zone in the minimum amount of time, emphasizing accessibility, and usability. We begin by presenting user data collected from our mobile application, *Entrain* [http://www.entrain.org], which recorded motion data and self-reported lighting histories for travelers crossing time zones [16]. Our results show: 1) predicted circadian phases for populations from five continents; 2) travelers flying west feel more favorable than those flying east; 3) people who more closely followed the optimal schedule feel more positive; and 4) mathematically optimal schedules are challenging to follow in real life. We then explore why travelers deviate from the optimal trajectory and the effects of these disruptions on the model. To correct the disruptions, we propose a novel method that recommends, in a dynamic way, an approximately optimal schedule from any point in the state space. Finally, we suggest several other ways to improve the practicality and usability of the recommended schedules. These methods can be extended to other models and can inform similar interventions targeting human behavior in the real world.

## Results and discussion

### Understanding real-world data

**Fig 1:**
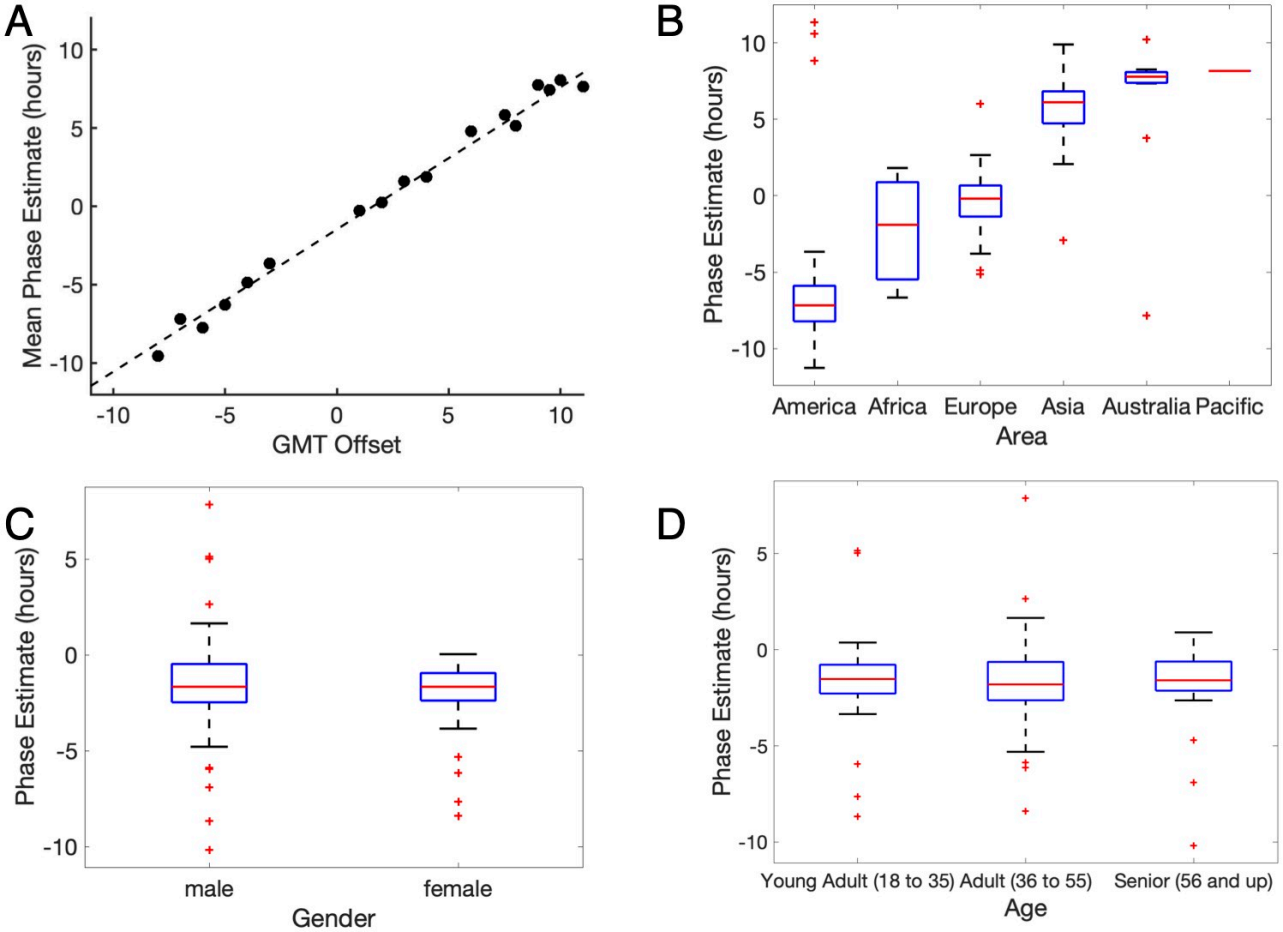
Phase estimates for users of *Entrain*. Motion data are processed through the mathematical model of the human circadian clock developed by Jewett et al. [5]. The sample includes 122 subjects with at least five consecutive data days and seven days with no travel. (A) Average phase estimates (in UTC) in different GMT Offsets. Each dot represents the average phase estimate for each GMT Offset. The dashed line shows the best-fit line to the data points across all GMT offsets. The best-fit line suggests the difference between phase estimates is consistent with the time zone difference. (B) Box-plot of phase estimates (in UTC) across continents. (C) Box-plot of phase estimates (in local time) between genders. (D) Box-plot of phase estimates (in local time) across different age categories.

Previous work has shown that activity coupled with mathematical models can predict circadian phase with a mean absolute error of approximately 1 hr for people living in normal conditions and an error of ~2.5 hr for non-rotated night shift workers [17]. Here, we applied the same mathematical model on motion data collected from the app *Entrain*. Fig 1 shows the predicted circadian phases for a sample of 122 *Entrain* users from five continents, where these 122 subjects have at least five days of consecutive data and seven days with no travel. Fig 1A shows the average of the predicted circadian phase of subjects in each time zone, and Fig 1B summarizes the distribution of circadian estimates for subjects in different continents. These results corroborate past research showing that activity can provide a coarse approximation of the circadian phase in the field setting [18], as the difference of phase estimates (in UTC) among time zones is consistent with the time zone difference. In addition, we find no statistically significant difference between genders (Fig 1C) (p-value = 0.32). As it is known that sleep is related to the circadian phase [19,20], our result is in line with previous research using the *Entrain* dataset that shows the difference between gender is not statistically significant in midsleep [16].

Regarding age, Fig 1D shows a trend that middle-aged adults have the earliest median predicted phase, which is 18 min earlier than the younger adults and 13 min earlier than the older adults. However, the difference is not statistically significant (p-value = 0.84). This difference is consistent with previous analysis of the *Entrain* data, which shows a quadratic trend in sleep with age, where middle-aged users were more likely to have shorter sleep duration and earlier wake times [16].

**Fig 2:**
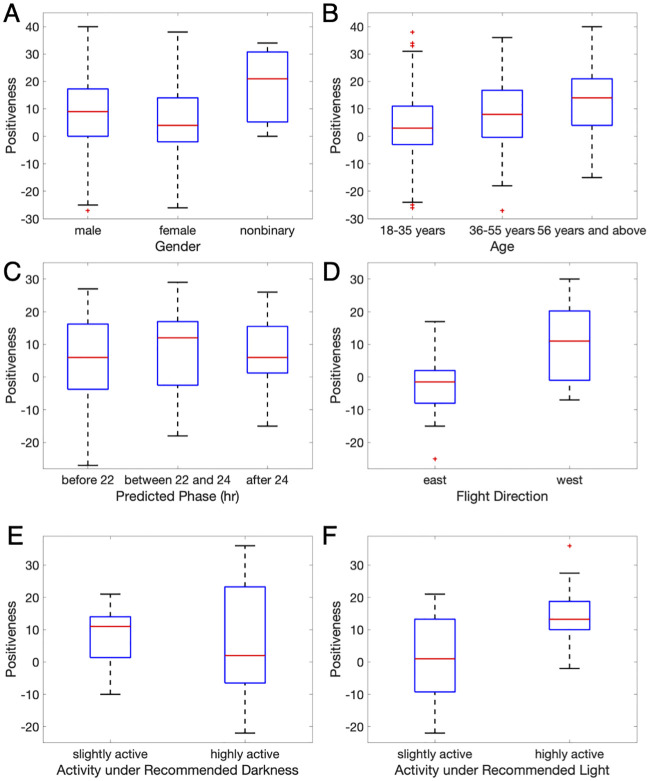
Mood assessments for users of *Entrain*. The mood assessment consists of 20 items, with ten items measuring positive affect and ten items measuring negative affect. Each item is rated on a five-point scale (1 indicating very slightly, five indicating extremely). The y-axis, ‘Positiveness,’ represents the difference between the ten items of positive affect and ten negative affect items. (A, B) A summary of mood assessments of 680 users completed within a week after trips shows results regarding gender (A) and age (B). (C) This figure shows mood assessments with predicted circadian phases. Seventy-one subjects submitted at least five consecutive days of motion data before filling out the mood assessment. A quadratic trend is observed in mood assessments with different phase estimates. (D) Thirty-one subjects completed mood assessments within one day after trips to assess mood for different traveling directions. This figure shows that jet lag, as measured by mood, is worse when flying east. (E, F) The relationship between subjects’ moods and the extent to which they followed their optimal schedule is shown. Twenty-eight subjects submitted their motion data during the recommended schedule for phase adjustments. For each episode of the recommended light schedule (i.e., avoiding/receiving light), the subject is considered slightly active if he/she is active under 30% of the time during the scheduled period, and highly active otherwise. This figure suggests that users who follow the recommended light schedules feel less jet lag.

The app *Entrain* is also equipped with a mood assessment, where the mood descriptor is taken from the Positive and Negative Affect Scale [21]. Subjects gauge their feelings via a questionnaire with 20 items: ten corresponding to positive affect and ten for negative affect. A 5-point scale is used to score each item. A summary of the mood surveys from 680 subjects that were completed within a week after a trip shows that men and older individuals report more positive affect (p-value < 0.01) (Fig 2A and 2B). Among the 680 subjects, 71 subjects submitted at least five consecutive days of motion data before filling out the mood assessment. A quadratic trend is shown in the median of mood assessments with predicted circadian phases. Subjects who have expected phases between 22:00 and 24:00 feel more positive (Fig 2C); however, the difference is not statistically significant (p-value = 0.83. Though how mood shifts throughout the trips are unknown, the mood assessments of 31 subjects who submitted their evaluation within 24 hours of their trip can reveal how recent jet lag affects mood. We find that jet lag, as measured by mood, is worse when people travel east (p-value = 0.02) (Fig 2D). This can be explained in part because the period of the human circadian pacemaker during free-running conditions is longer than 24 hours [22]. It is more difficult to phase advance the human circadian clock than to phase delay it [23,24].

To validate the hypothesis that the optimal schedule corrects circadian misalignment, we use the phone-recorded activity to measure how well the optimal schedule is being followed. People who follow the optimal schedules are likely to be more active when light exposure is recommended and inactive when darkness is recommended. For each light or dark block from the recommended optimal schedules (i.e., receiving/avoiding light), the subject is considered highly active if the subject remains active more than 30% of the time during the scheduled period, and slightly active otherwise. We find that people who better follow the optimal schedule (i.e., less active under recommended darkness and more active under recommended light) are significantly more able to overcome jet lag (p-value = 0.02), at least as measured by mood (Fig 2E and 2F).

**Fig 3:**
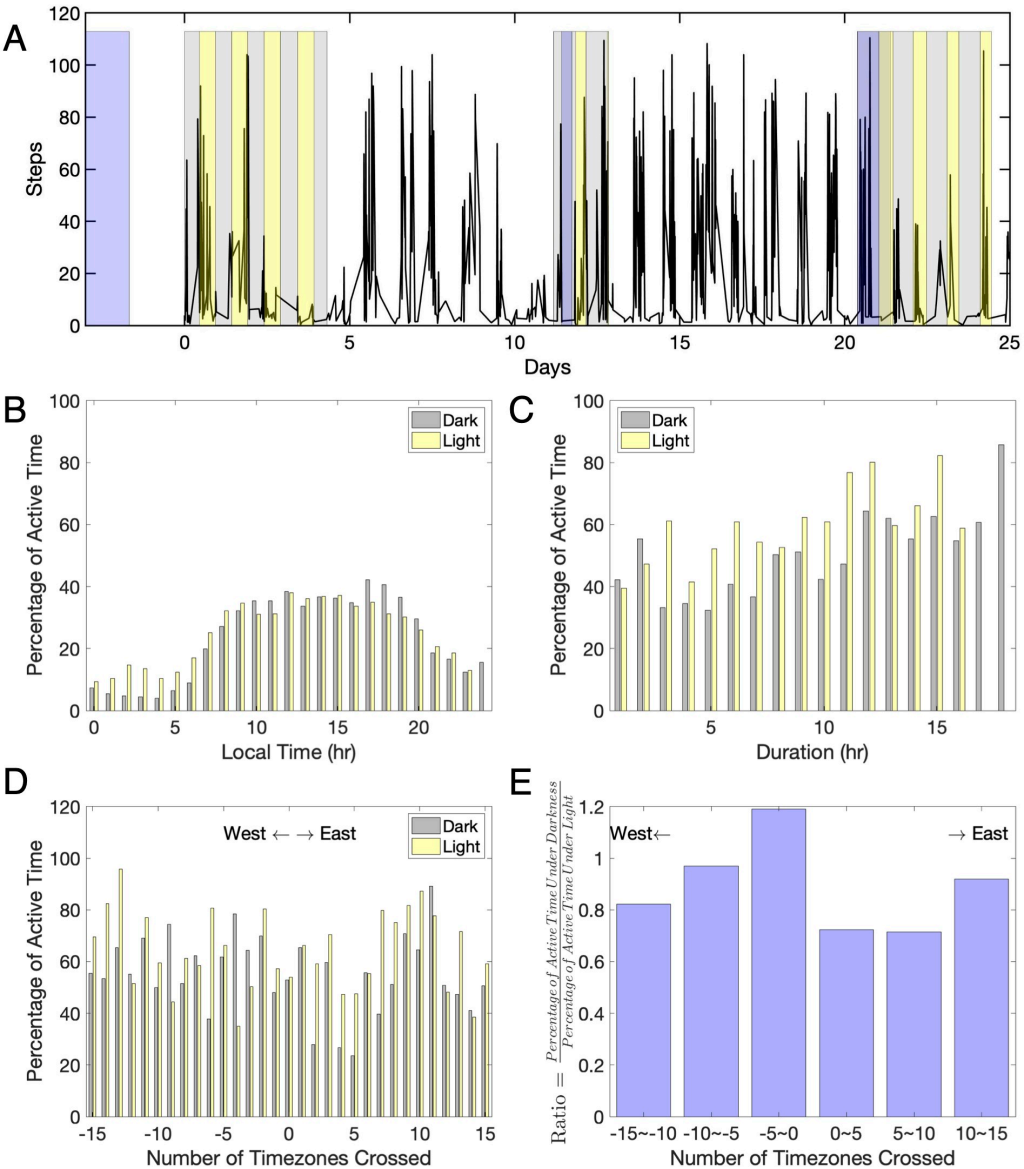
Optimal schedule deployed in real life. (A) Activity data (black line) from one subject who took three trips (shaded in blue) in 25 days. Optimal schedules (grey for avoiding light, and yellow for receiving light) to adjust to the new time zones were computed for each trip. We can see the subject generally followed the recommended optimal schedule for his/her first and last trip, but not for the second trip. (B) A sample of 147 *Entrain* users who submitted their motion data during trips shows how the recommended schedules were followed each hour of the day. Each yellow/gray bar represents the average percentage of active time during the scheduled light/dark period at each hour of the day. Unsurprisingly, regardless of the recommended light-receiving/light-avoiding schedule, the activity pattern peaks during the day and drops during the night. (C) This figure shows the extent to which the subjects followed the recommended schedule for different time durations. Each yellow/gray bar represents the average percentage of active time at each recommended duration of the light/dark period. As the recommended period of darkness increases, so does the percentage of active time under recommended darkness. (D) This figure shows how the recommended schedules were followed as subjects traveled across different time zones, where positive time zones represent traveling east. Each yellow/gray bar represents the average percentage of active time during the recommended light/dark period for the total number of time zones crossed. (E) This figure shows the percentage of active time under recommended darkness under the recommended light for the total number of time zones crossed. As can be seen, the optimal schedule is harder to follow when flying west on short trips. The low compliance with the schedule may also occur because people do not feel the need to observe light and dark recommendations for short phase delays.

Time-optimal schedules for shifting the clock, like those computed in Serkh and Forger [10], can prove challenging to follow under real-world conditions. Fig 3A demonstrates one *Entrain* user’s motion data during three trips, which shows that this subject roughly followed the recommended schedule for the first and third trip but not for the second trip. An analysis of the entire sample of 147 subjects who submitted their motion data during trips suggests that people rarely follow the recommended light schedule. Fig 3B shows that the activity pattern peaks during the day and drops during the night, regardless of the recommended schedule for light or dark. Moreover, Fig 3C demonstrates that the percentage of active time under recommended darkness increases as the recommended period of darkness increases. This suggests that regardless of the recommendation of avoiding light, subjects generally spend a limited amount of time under darkness, as avoiding light for long hours is not practical in the real world.

Interestingly, we find that when flying west (e.g., flying from New York to California), subjects tend to be more active under recommended darkness (Fig 3D and 3E). This is likely because the optimal schedule for a short westward trip can recommend avoiding light until late afternoon (e.g., 5 pm) in the destination time zone. This is a difficult-to-follow recommendation for many people and one that, combined with the relative ease of making a short phase delay without any lighting recommendations, appears to have often had low compliance.

### Recovering from disruptions

Data from the app, *Entrain*, have shown that following optimal schedules correlates with a more positive reported mood in travelers. We have also demonstrated that travelers often fail to follow the optimal schedules precisely in the real world. This is a problem inherent to travel: people on trips are likely to deviate from recommended schedules in unexpected ways, causing their circadian state to deviate from the original optimal trajectory and invalidating the initial recommendations.

**Fig 4:**
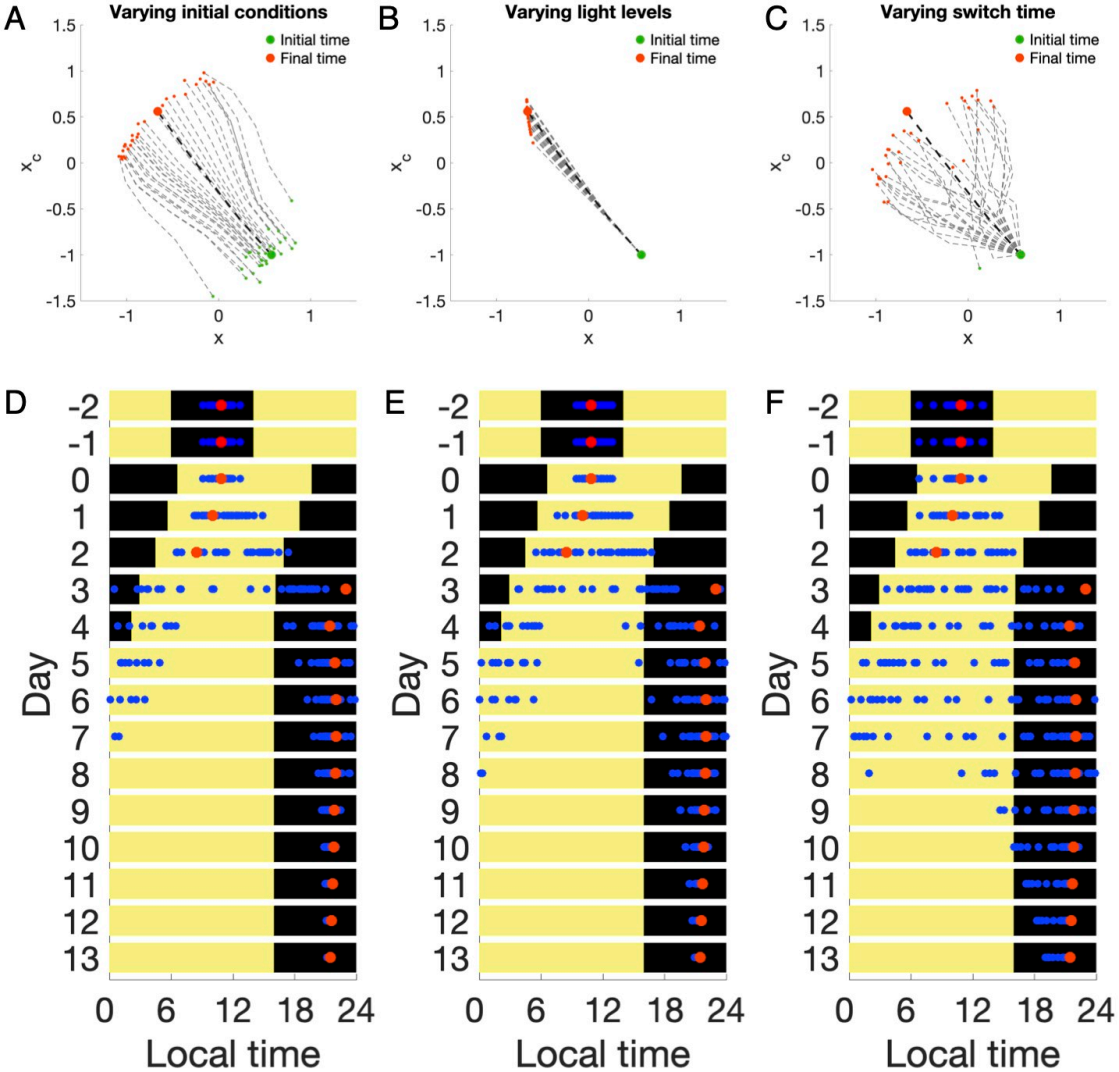
Sources of error. Optimal schedules for entrainment to 12-hr shifts are simulated with different types of noise for 30 hypothetical subjects. (A-C) Phase trajectories with other sources of error were individually studied. The human circadian pacemaker acts as a limit cycle oscillator formed by two variables, x, and xc. The variable x represents the core body temperature, a phase marker of the human circadian rhythm, and the variable xc is required to achieve a limit cycle mathematically. The noiseless, optimal trajectory is plotted in the dark dashed line, while noisy trajectories are plotted in a lighter gray. The start and end of the trajectories are marked in green and red, respectively. Three sources of error were considered: initial conditions (i.e., starting circadian state), light levels in lux, and switch time (the times at which light is either switched on or off). For each source of error, the noise was sampled randomly from a normal distribution with mean 0 and standard deviation σ, where the standard deviation in each case is *σ*_initial condition_ = (*σ*_amplitude_, *σ*_phase_) = (0.1,1), *σ*_light_ = 1000 lux, and *σ*_switch time_ = 2 hours respectively. (D-F) 24-hour snapshots of the circadian phase with all different types of noise acting together. Graphs from left to right represent the increasing magnitude of noise added to switch times (*σ*_switch time_ = 2, 3, 4 hours), with a fixed magnitude of noise added to initial conditions and light levels *σ*_initial condition_ = (*σ*_amplitude_, *σ*_phase_) = (0.1,1), *σ*_light_ = 1000 lux). Predicted core body temperature minima (CBTmin) are plotted against the schedule of optimal light exposure, where the color yellow and black represent bright light exposure (10000 lux) and darkness (0 lux), respectively. Predicted CBTmin under the optimal schedule without noise is marked in red circles, while predicted CBTmin of 30 hypothetical subjects under schedule with noise is plotted in blue circles.

Many such deviations affect the process of adjustment (entrainment) along an optimal trajectory predicted by a model, including inexact levels of maximum light exposure, inaccurate knowledge of the correct switch times (when the traveler started and stopped seeking light exposure), and unknowns about the user’s starting circadian state. Fig 4 illustrates how each noise source affects the optimal schedules for 30 hypothetical subjects to complete entrainment in minimum time to a 12-hour phase advance. For each source of noise and each simulated subject, the noise was sampled from *N*(0, σ^2^), i.e., a normal distribution with mean 0 and standard deviation *σ*. The standard deviation in each case is *σ*_initial condition_ = (*σ*_amplitude_, *σ*_phase_) = (0.1,1) (Fig 4A), *σ*_light_ = 1000 lux (Fig 4B), and *σ*_switch time_ = 2 hours (Fig 4C), respectively. We find that variations in initial conditions and switching times have the most significant impact on entrainment. However, varying light levels from 3000 lux do not significantly affect the phase shifts (Fig 4A–4C). We then tested the optimal schedule with all types of noise acting together, with varying degrees of bias in switch times (*σ*_switch time_ = 2, 3, 4 hours). Fig 4D–4F shows that the optimal schedule entrainment holds when the standard deviation of the noise is relatively small (Fig 4D and 4E). However, large deviations from the optimal trajectory can be seen if the standard deviation in switch times is increased to *σ*_switch time_ = 4 hours, as in Fig 4F, and this amount of departure from the original schedule, while significant, could very easily occur under the time pressures of travel.

In light of this, it is necessary to develop tools that can recommend schedules dynamically to correct the large deviations from the original schedule encountered in the real world. The Switch Time Optimization algorithm is too computationally costly to recompute recommendations for every departure from the initial schedule. Therefore, we propose an "approximately optimal" schedule, which is not exactly the optimal schedule but is less expensive to compute and still drives the system to the target time zone’s limit cycle.

**Fig 5:**
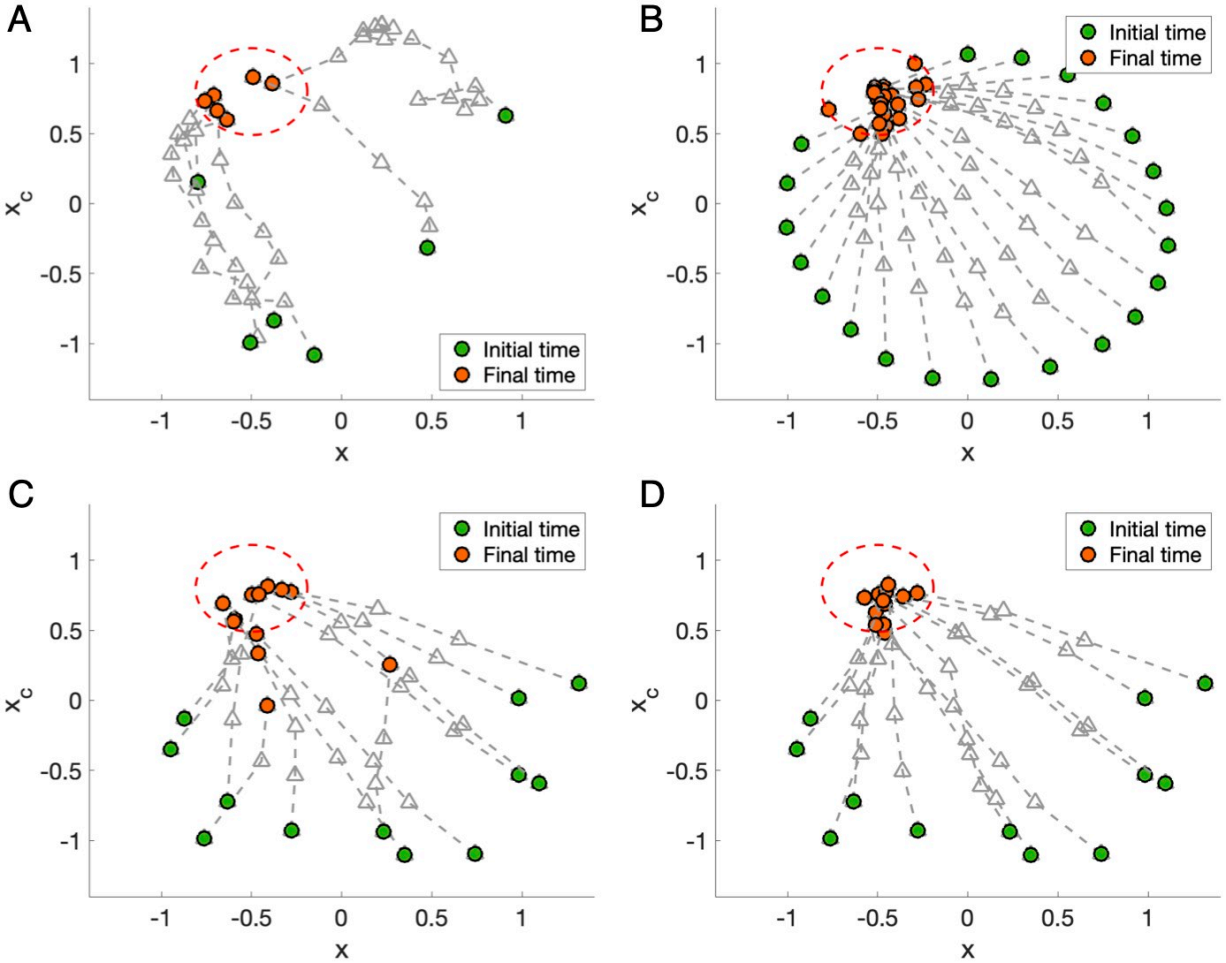
Simulated trajectories from real data, optimal schedule, and nearest optimal schedule. To illustrate how optimal schedules and closest optimal schedules work, all four figures here select a target zone (dashed red circle) in the upper left quadrant. The target zone represents the point at which a trajectory is within a tolerance of the perfectly entrained target point. The variables, x, and x_c_, form a limit cycle, representing the human circadian pacemaker with 24.2 hours. The predicted circadian phase of each day is marked with a triangle (Δ). Green dots mark the start of trajectories, and the end of trajectories after sufficient entrainment are marked in red. (A) Sample trajectories are simulated by the self-reported lighting history of *Entrain* users with the circadian model. As can be seen, by the number of triangles and the trajectories’ winding direction, phase shifting in the real world is inefficient. (B) Sample trajectories of optimal schedules starting from 24 initial states that are evenly sampled on the limit cycle are plotted in the (x, x_c_) space. We can observe that the optimal trajectories are almost straight lines, indicating that the entrainment is efficient. (C) Trajectories follow the nearest optimal schedule from randomly selected starting points. A subset of twelve starting points is randomly chosen from the starting points on the limit cycle in (B) added with noise. The noise is randomly sampled from a normal distribution with mean 0.5 and standard deviation 0.25. Following the nearest optimal schedule introduces error, which results in some final states not arriving in the target zone. (D) Trajectories follow the nearest optimal schedule from randomly selected starting points with schedule updates occurring at regular intervals by repeating the method of the nearest optimal schedule. As can be seen, updating the schedule at regular intervals corrects the error and results in all final states landing in the target zone.

Intuitively, shifting phase from one state to another with optimal schedules would occur via a straight-line trajectory, where the distance of the trajectory in phase space is minimized. Fig 5A shows that phase shifting without optimal control is inefficient. The trajectories simulated from self-reported lighting history have multiple detours and take longer (each triangle represents a day) to reach the final target state. Interestingly, we also observe that to reach a fixed target time zone, the trajectories following the optimal schedules from different starting points on the limit cycle cover most of the (x, x_c_) state space (Fig 5B). In this state space, x is shifted so that its minimum occurs at the same time as the human core body temperature rhythm, and xc is mathematically required to achieve a limit cycle to describe the human circadian pacemaker [4,5]. Given any starting point in (x, x_c_) space, we can approximate the optimal trajectory to the target time zone by taking and modifying the switch time from the nearest optimal trajectory starting from a point on the limit cycle that passes by our starting point.

Suppose, given a target time zone, we seek an optimal schedule that begins at the point $(x^{*},x_{c}^{*}$) at time t* (for instance, starting from any of the green markers in Fig 5C and 5D). Such a schedule can be approximated in the following way:

1. Compute and store values for (*t*, *x*, *x*_*c*_, *n*) for the optimal trajectories that begin on the limit cycle, with *t* ∈ [0, *t*_*f*_] sampled with a timestep *dt*.
2. Interpolate over each pre-computed trajectory to estimate the set P of all points $\left(\hat{t},\hat{x},\hat{x_{c}},\hat{n}\right)$ such that $\hat{t}\equiv t^{*}$(mod 24).
3. Select the point $\hat{x}$ in P that minimizes $\left|x^{*}-\hat{x}\right|$ where $x^{*}=(x^{*},x_{c}^{*},n^{*}$) and $\hat{x}=(\hat{x},\hat{x_{c}},\hat{n})$
4. Shift the switch times from the pre-computed optimal schedule corresponding to the selected point $\hat{x}$ by subtracting $\hat{t}$ by subtracting and discarding negative values.
5. (Optional) Repeat Steps 2–3 at regular intervals between $\hat{t}$ and entrainment *t*_*f*_, selecting a different point $\hat{x}$ and schedule if one is closer to the new trajectory than the previous choice of the nearest optimal schedule.
6. (Optional) Run a small number of iterations of the original Switch Time Optimization algorithm to refine the output.

Intuitively, following the nearest optimal schedule will introduce error. The end of the trajectory will likely not be within the same tolerance of the target time zone as the original schedule (Fig 5C). However, re-calibrating the schedule by repeating the process of recommending the nearest schedules at later time points can prevent such error from accumulating (the optional step in the method above). Compared to Fig 5C, the end of the trajectories in Fig 5D are more clustered and stay within the target time zone’s tolerance when the schedule is updated repeatedly. Though the nearest optimal schedule is not exactly the optimal schedule, it will still drive the system closer to the limit cycle. The repeated corrections to the schedule at regular intervals can steer trajectories back on course. More importantly, the nearest optimal schedule can be adjusted and updated dynamically in response to the variations of disruptions.

### Improving compliance

#### Limiting time spent in light and dark

Recommending schedules that respond dynamically to noise is one option for improving the usability of optimal schedules. Another alternative is to increase the likelihood that users will be able to follow the originally recommended schedules.

Past work in the computation of optimal schedules for achieving circadian shifts did not limit the amount of time spent in light and dark each day. Though this is not a concern in many cases, it can lead to schedules with intensively long periods of darkness or light, which are almost impossible to follow. As referenced in the previous section, in Serkh and Forger [10], the optimal schedule for a three-hour phase delay required users to stay under darkness for 25 hours out of a total adjustment time of 32 hours. However, for most people who attempt to phase delay three hours, it is not worth spending such long periods of darkness improving their entrainment speed.

Therefore, to avoid extended periods of recommended light and dark and to improve the utility of the recommendations, we recomputed the optimal schedules with the constraints that the blocks of times spent in the light must be greater than five hours in duration and the blocks of times in dark must be less than ten hours. These constraints were chosen so that the recommended schedules still provide fast entrainment while also being more practical to follow (more details are discussed in Methods).

**Fig 6:**
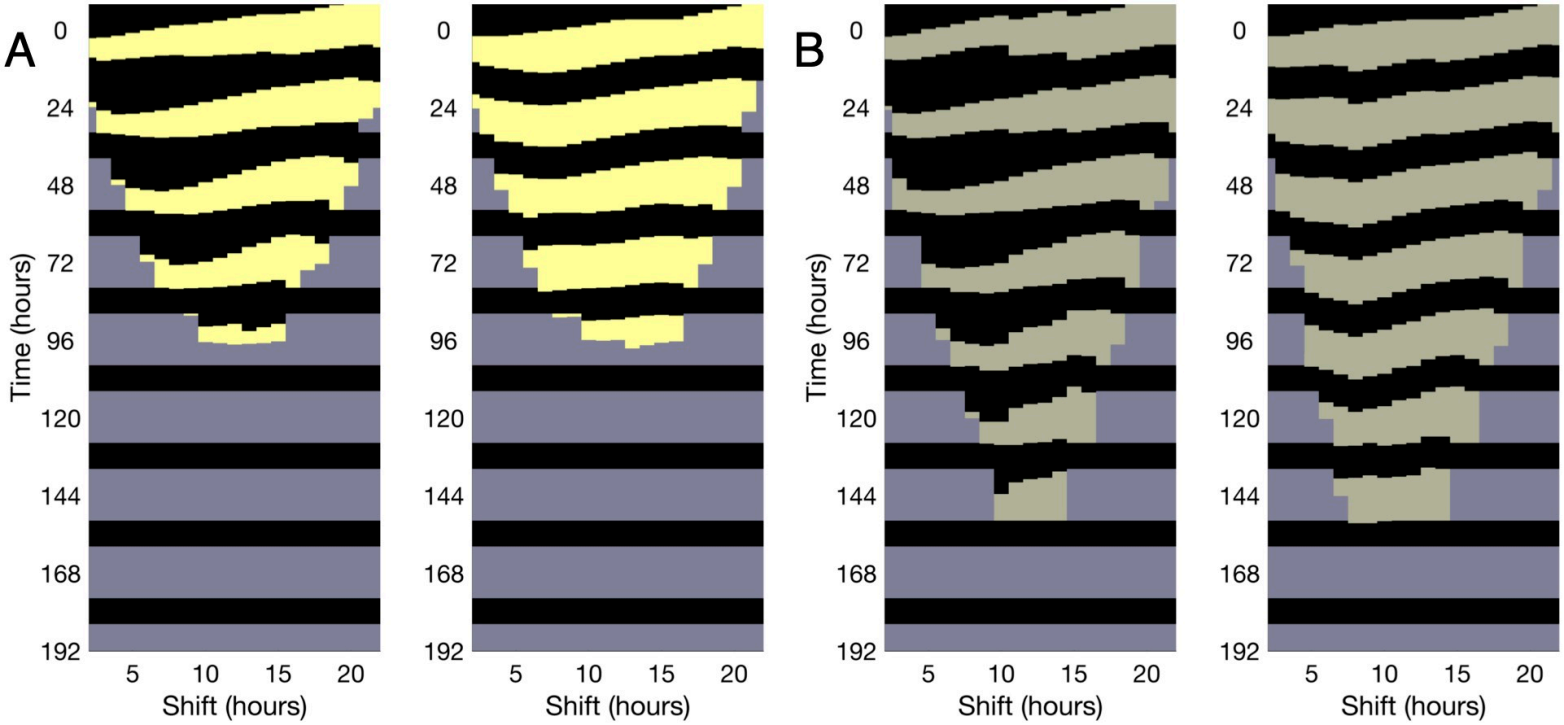
Comparison of optimal and accessible lighting schedules for different light levels. (A) (Left) Optimal light schedules are computed with no constraints on light/dark duration, with a maximum lux value of 3000 lux. (Right) Optimal light schedules are calculated with constraints, where we require the times spent in light to be greater than five hours in duration and the times in dark to be less than ten hours. Each vertical slice of light and darkness is a schedule, with the colors defaulting to the background gray when a person following the schedule is sufficiently close to entrained. The time to entrainment is nearly identical despite markedly different durations of darkness for some shifts. (B) (Left and Right) Unconstrained and constrained optimal schedules for a maximum lux value of 500 lux.

Fig 6A(left) and 6B(left) shows the solutions to the minimum time phase shift problem in the absence of any constraints, which agrees with the results obtained in Serkh and Forger [10]. As in [10], phase delaying schedules show long periods of darkness, continuing into the new time zone’s daylight hours. If the constraints on the times spent in light/dark are imposed (Fig 6A(right) and 6B(right)), we find that it takes approximately the same amount of time to become entrained as the unconstrained solutions. In general, the bound on maximum darkness is reached more often than the bound on minimum light, and these constraints affect the phase delaying adjustments with long periods of darkness the most. However, even in the most dramatically altered schedules, the time it takes to entrain increases by less than 14 hours (with a total time of adjustment up to approximately 90 hours).

Furthermore, while the previous work in Serkh and Forger [10] only computed schedules that began at the start of light in the target time zone, we have extended this computation for schedules that can start from any hour of the day. No significant differences in entrainment time across schedules were found, which suggests that choosing the starting time of a schedule does not significantly enhance or harm entrainment speed.

### Stopping an optimal schedule mid-adjustment

Longer-term compliance is a concern even for essential interventions, and jet lag schedules that take too long to entrain have the risk of being swiftly ignored. For example, scheduling activity around the recommended light and dark times may not seem valuable by day three or four of an adjustment. Travelers may opt to revert to a “slam shift” adjustment, that is, returning to their regular light/dark schedule in the new time zone (where “slam shift” means an abrupt shift in the light/dark cycle to the new time zone).

To understand how this affects entrainment, we simulated to find the point in the adjustment process, at which slam shifting and following the optimal path become nearly indistinguishable. Calculating and conveying this point of “slam equivalence,” past which the optimal schedule and slam shifting entrain in almost the same time, could help the travelers who consider stopping the schedule early to persist until most of the speed benefits are gained from the optimal path.

Here, we computed the average phase difference between the final point of the prematurely ended optimal schedule and the target over the next 24 hours as a function of how much of the optimal schedule has been followed. We assume that any time past the halting point reverts to the slam shift schedule, i.e., the regular light schedule, in the target time zone.

**Fig 7:**
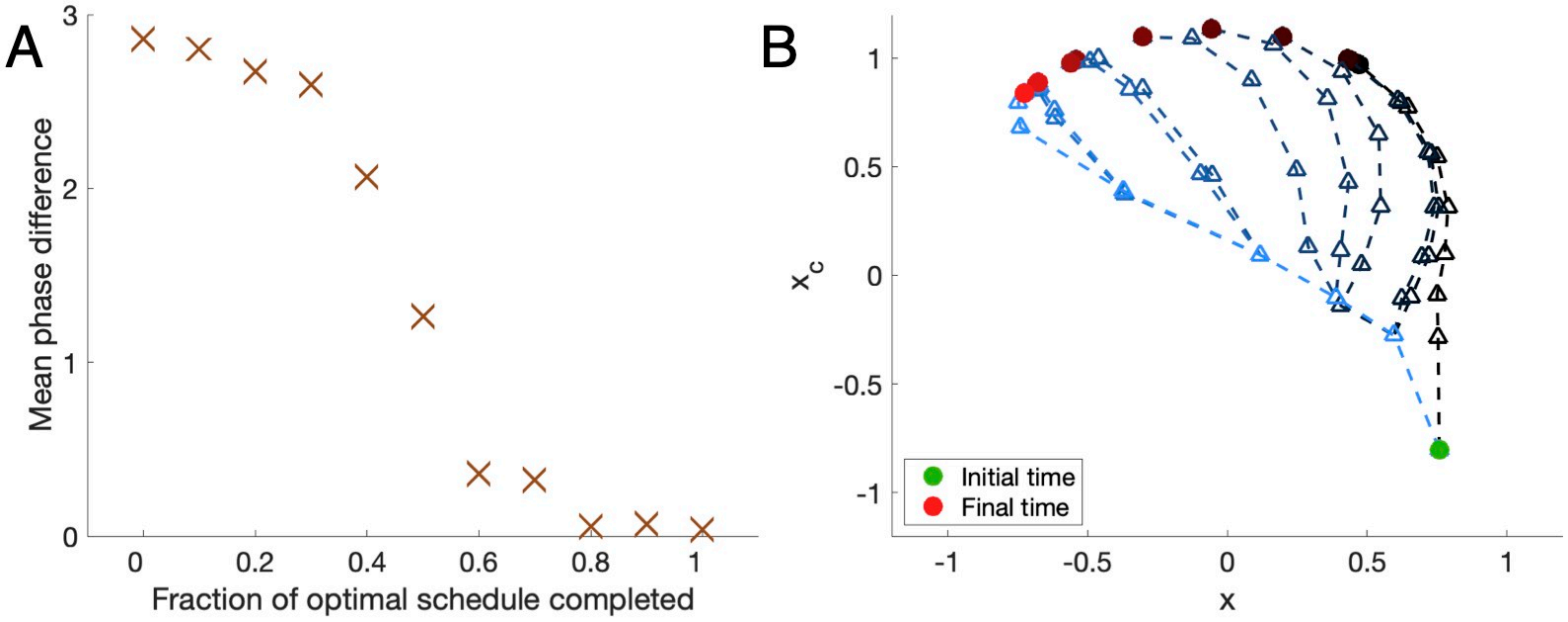
Effect of stopping an optimal schedule early. (A) Mean phase difference between the final point of the prematurely ended the optimal schedule and the target over the next 24 hours. The function is nonlinear, with a sharp change in the mean phase difference (and time to entrainment) occurring at approximately the halfway point in the schedule. The final difference is non-zero due to the tolerances set for convergence in the Switch Time Optimization algorithm [25]. (B) Visualizing the trajectories by sampling every 24 hours in phase space. The green circle marks the starting point. Lighter trajectories with brighter red circles as the final states correspond to more of the optimal schedule completed. A triangle marks each day’s predicted phase.

In an example of a 12-hour shift, the mean phase difference function is nonlinear (Fig 7). In particular, it shows that after two-thirds of the optimal schedule has been completed, the slam shift and optimal schedules are very similar in their ability to push the clock’s phase towards the target time zone. On the other hand, travelers who only follow the first third of the optimal schedule experience almost none of the optimal schedule’s speed improvements.

While only one 12-hour shift was examined in Fig 7, the following property is applied to almost all other shifts: within some radius of the target limit cycle, slam shift is nearly as effective at phase shifting as the optimal trajectory. Practically, this implies that users traveling across time zones could be informed of the point at which they are "slam equivalent" or within the near locus to entrainment in which slam shift is as effective at phase shifting as the optimal schedule. This could reduce the time spent in following an optimal recommendation by as much as a half. While dropping from the optimal schedule and assuming a slam shift schedule is, by definition, sub-optimal, the benefits from schedule flexibility and ease of use could easily outweigh the longer time to entrainment for some users.

## Conclusion

Here, we consider the practicality of schedules previously proposed to overcome jet lag in minimal time [10]. These schedules were presented to travelers in a smartphone app *Entrain* that also recorded users’ activity, which could determine how well the users followed the schedule. We find that users are rarely able to achieve perfect adherence to the recommendations. Better adherence to the proposed schedules yields better outcomes, at least as measured by positive affect. Taken together, it suggests that a more practical approach for phase shifting in the real world would be useful.

We then sought to improve optimal schedule recommendations in light of what we have learned from the *Entrain* app users. We consider three potential sources of variability between individuals. Interestingly, variations in light level, which could occur for many reasons (e.g., angle of gaze or cloud cover), which would be very difficult to control, do not significantly affect the ability to phase shift quickly. The ability to accurately assess the initial state of a user’s circadian clock is an essential factor in the ability to phase shift them. Besides initial conditions, changes when light is started or stopped also remain an essential factor to affect phase shifting.

We find that not all parts of a schedule are equally crucial for adjusting to a new time zone. The earlier parts of a schedule can be more important than the later parts; stopping the schedule two-thirds of the way can yield almost as much benefit as completing the schedule. Likewise, some impractical aspects of the schedules can be ignored, such as long periods of darkness, to minimal effect. We propose that future work focus on practicality rather than minor improvements in adjustment time.

It is also helpful to compare our work to previous computational attempts to determine optimal schedules. Another approach to the work we present here would be to simplify the mathematical models we use. Most papers in the literature propose a static schedule recommendation; however, we find that mathematical models can personalize and greatly enhance the effectiveness of a schedule. While we use the original model proposed by Serkh and Forger [10], optimal schedules from simpler models can sometimes be used to achieve similar entrainment times, as shown in an extensive study by Julius et al. [9]. However, it remains to examine whether these simplifications yield different or similar schedules. Finally, the *Entrain* app interpolates between schedules using the method we propose here. A similar feedback controller using Neural Networks to interpolate was studied by Julius et al. [9], where it shows that our assumptions of not interpolating the initial value of the *n* variable (that presents phototransduction) nor the maximal light level used as a constraint did not affect the performance.

One limitation of our analysis regarding the Entrain app is that the recorded activity is considered a proxy to light to estimate the circadian phase and examine how well users follow the optimal schedules. In particular, though individuals are more likely to be in a dim environment, the possible errors, such as staying inactive in a bright environment (e.g., lying on the beach) and wearing sunglasses, cannot be reflected from activity. Moreover, the initial conditions used to predict the circadian phase for *Entrain* users are determined by the self-report light/dark histories, resulting in systematic errors in the phase estimates. Also, we only consider the effect of white light in our study. Optimal schedules regarding the use of other spectral components of light (e.g., blue light) can be considered in the future [26]. Nevertheless, we have been able to use mobile technology to collect massive data at virtually no cost, and advancing technology and wearables available to the public will enable us to gain further insights into circadian timekeeping in the real world.

This work is essential in moving basic science results about circadian timekeeping to the clinic and the real-world. We have shown that users can benefit from schedule recommendations when crossing time zones. The user can modify some aspects of these schedules without significantly changing the ability to adjust to a new time zone. As real-world constraints can be unpredictable, it is essential that schedules can be adjusted, and such adjustments can be achieved using the methods we proposed above. We present a new paradigm in changing human behavior: putting users first, where personalized recommendations are determined from mathematical models, and as real-world constraints occur, new recommendations are calculated. This paradigm is commonly used for GPS navigation. It has now been tested to overcome jet lag, which sets the stage to apply the same paradigm to be used in many other interventions for human behavior in the future.

## Methods

### Ethics statement

All data collected by Entrain are anonymous. The University of Michigan Health Sciences and Behavioral Sciences Internal Review Board determined that this research was exempt from ongoing IRB review and approval.

### Data

The Entrain app relays mathematically optimal light and dark schedules to help users shift time zones as quickly as possible. The app collected steps and heart rate data from users with Apple Watch, who opted-in and self-report light and dark histories. The app also includes a reaction time assessment and the Positive and Negative Affect Scale (PANAS). All data was transmitted anonymously, and users were able to opt-out of data collection at any time. More details of Entrain is available in the previous work [16] and the website (https://www.entrain.org). A total of 8254 users have opted in to submit their data for Entrain (version 3).

A total of 3317 subjects uploaded their motion data from Apple Watch, where 122 subjects uploaded five consecutive days of data with no travel in a week (S1 Table). 823 subjects filled out the mood assessment and the demographics questionnaire. Among these 823 subjects, 680 subjects submitted their mood assessment within one week after trips (S2 Table), and 31 subjects completed it within 24 hours after trips. A total of 147 subjects submitted their motion data during the recommended schedule for phase re-entrainment, and 28 of them submitted a mood assessment.

### Initial conditions

Self-report light and dark histories are used to determine the initial conditions to simulate the human circadian clock model.

### Statistical analysis

Statistical analysis was performed in Matlab (Mathworks; version R2019a). The Wilcoxon rank-sum test was used to test whether the two groups were significantly different from each other. The Kruskal-Wallis test was used when there are more than two groups.

### Solving a minimum time problem

As in Serkh and Forger [10], the primary algorithm used to compute the optimal schedules is the Switch Time Optimization (STO) method, first described in [25]. The advantage of this algorithm is its ability to work well with a system of state equations

$$\dot{\text{x}}=f(x,\;u)$$

The optimal control is “bang-bang”, meaning that the optimal control only takes values on the boundary of its allowed values. The method iteratively updates a set of “switch times” *t*_*j*_, the points at which the control moves from its lowest to highest allowed values (darkness to max lux) or vice versa in order to minimize some cost function. Our cost function has two constraints: the final time t_f_ must be minimized and final position must be on our target circadian phase (*x*(*t*_*f*_) = *x*_*target*_(*t*_*f*_)).

Our goal is to minimize the function in terms of the final time

$$J=t_{f}$$ (1)

While maintaining the dynamic constraint x(t_f_) = x_target_, which is enforced by the equation ψ(t_f_) = |x(t_f_) = x_target_(t_f_)|^2^ = 0. The dynamic constraint is adjoined to the cost function by adding the vector of Lagrange multipliers *R*, and the new cost function is defined by

$${\text{J}}^{2}={\text{t}}_{\text{f}}+\nu^{\text{T}}\psi ({\text{t}}_{\text{f}})+\int_{{\text{t}}_{0}}^{{\text{t}}_{\text{f}}}{\text{R}}^{\text{T}}(\text{f}(\tilde{\text{x}},\text{u})-\dot{\text{x}})\text{dt}$$ (2)

where *ν* is a positive scalar chosen to enforce the dynamic constraint.

Our Goal is to calculate *ν* and *R* so that the changes dt_f_ and dt_i_ decrease the value of *J*^2^.

The steps of the algorithm are described as follows:

1. **Initialize**. Start with an initial guess for the final time to entrainment, *t*_*f*_, and the set of switch times, *t*_*j*_ ∈ [0, *t*_*f*_]. From these switch times, build the control function u(t) ∈ [u_min_, u_max_]. (In the case of circadian control, *u*_min_ is zero and *u*_max_ is the brightest light level in lux available to the system)
2. **Integrate system forward**. Fix *x*(0) to be the circadian phase at the initial time (henceforth indexed as t = 0) and integrate the system of equations $\dot{x}=f(x,u)$ forward in time.
3. **Integrate sensitivity functions backwards**. The Sensitivity Functions *R* are defined to be the solutions of the following differential equation.
  $${\text{R}}^{\prime }=-{\text{R}}^{\text{T}}\frac{\partial \text{f}}{\partial \text{x}}(\text{t})$$
  $$\text{R}({\text{t}}_{\text{f}})=\nu^{\text{T}}\frac{\partial \psi }{\partial \text{x}}(t_{f})$$
  Note that here we are integrating backwards in time from *t* = *t*_*f*_ to *t* = 0 with *x* values from the path calculated in step 2.
4. **Update the switch times**. It can be shown that
  $$\text{d}{\text{J}}^{2}={\left(1+\nu^{\text{T}}\frac{\partial \psi }{\partial \text{t}}+{\text{R}}^{\text{T}}\dot{\text{x}}\right)}_{\text{t}={\text{t}}_{\text{f}}}\text{d}{\text{t}}_{\text{f}}+\int_{{\text{t}}_{0}}^{{\text{t}}_{\text{f}}}{\text{R}}^{\text{T}}\frac{\partial \text{f}}{\partial \text{u}}\delta \text{u}\;\text{dt}$$ (3)
  for corresponding changes in control *du* and final time *dt*_*f*_.
  Since we are only considering “bang bang” changes at the switch times *t*_*i*_, this is equivalent to
  $$\text{d}{\text{J}}^{2}={\left(1+\nu^{\text{T}}\frac{\partial \psi }{\partial \text{t}}+{\text{R}}^{\text{T}}\dot{\text{x}}\right)}_{\text{t}={\text{t}}_{\text{f}}}\text{d}{\text{t}}_{\text{f}}++\sum_{i=1}^{m}{\left(R^{T}\frac{\partial f}{\partial u}\delta u(t)dt_{i}\right)}_{t=t_{i}}$$ (4)
  Let
  $$\text{d}{\text{t}}_{\text{f}}=-\frac{1}{\text{b}}{\left(1+\nu^{\text{T}}\frac{\text{d}\psi }{\text{dt}}+{\text{R}}^{\text{T}}\dot{\text{x}}\right)}_{\text{t}=\text{tf}}$$ (5)
  and
  $$\text{d}{\text{t}}_{\text{i}}=-{\left(\frac{{\text{W}}_{\text{ii}}^{-1}}{\delta \text{u}(\text{t})}\frac{\partial \text{H}}{\partial \text{u}}\right)}_{\text{t}={\text{t}}_{\text{i}}}$$ (6)
  where *b* is a positive constant, *W* is a positive definite weighing matrix, *H* is the Hamiltonian matrix *R*^*T*^*f*(*x*(*t*), *u*(*t*)) and *ν* is chosen to make ψ(*t*_*f*_) go to 0. A simple calculation can show that these changes of steps will drive the cost function (Eq (4)) to 0.
5. **Repeat**. Return to Step 2 and repeat the process with the new *t*_*f*_ and new switch times.

For more information on this algorithm, see [27].

### Constraining time spent in light and dark

Constraining the amount of time spent in each period of light and darkness to a given range requires us to modify the Meier-Bryson algorithm described above. Let *M* and *m* represent the maximum and minimum numbers of hours, respectively, where *M* and *m* can exist between two consecutive switch times. We will refer *M* and *m* as the maximum and minimum switch time distances. Note that without a prescribed minimum distance, the schedules would collapse to the unconstrained case, with infinitely fast switches segmenting any block of light or darkness exceeding *M* in duration.

As in the original algorithm, switch times are updated in a way that drives the system to a local minimum, with the added condition that schedules on the boundary of our space of acceptable functions may only be updated in ways that do not cause any switch time distance to leave the range [*m*, *M*].

For a control with at least one switch time *t*_*j*_ satisfying *t*_*j*+1_ − *t*_*j*_ = *M*, only switch time updates for which *dt*_*j*_ = *dt*_*j*+1_ are valid; the same is true for *t*_*j*_ satisfying *t*_*j*+1_ − *t*_*j*_ = *m*. Substituting this into Eq (6), we see that simply adding the weighted rate of change in the Hamiltonian for switch times *t*_*j*_ and *t*_*j*+1_ gives us our effective rate of change in the Hamiltonian for changing both switch times, thus the choice of perturbation $\hat{dt_{j}}$ that drives the cost to zero while maintaining the duration constraints becomes

$$\hat{dt_{j}}=dt_{j}+dt_{j+1}$$

In this way, the two switch times on the boundary are "glued" together and, as such, update and shift by the same amount.

## Supporting information

S1 Table Table of 122 subjects who submitted at least five consecutive days of motion data with no travel in a week. (XLSX)

S2 Table Table of 680 subjects who filled out the mood assessments within one week after trips. (XLSX)
